# Treatment and molecular profiling of acrodermatitis continua of Hallopeau during pregnancy using targeted therapy

**DOI:** 10.1016/j.jdcr.2021.09.002

**Published:** 2021-09-08

**Authors:** Sara Al-Khawaga, Roopesh Krishnankutty, Gulab Sher, Khairunnisa Hussain, Joerg Buddenkotte, Martin Steinhoff

**Affiliations:** a Department of Dermatology and Venereology, Hamad Medical Corporation, Doha, Qatar; b Translational Research Institute, Academic Health System, Hamad Medical Corporation, Doha, Qatar; c Dermatology Institute, Academic Health System, Hamad Medical Corporation, Doha, Qatar; d Department of Dermatology, Weill Cornell Medicine, New York, New York; e Qatar University, College of Medicine, Doha, Qatar; f Weill Cornell Medicine-Qatar, College of Medicine, Doha, Qatar

**Keywords:** guselkumab, certolizumab, cytokine profiling, psoriasis, proteomics, ACH, acrodermatitis continua of Hallopeau, IL, interleukin, TNF, tumor necrosis factor

## Introduction

The etiology and pathogenesis of acrodermatitis continua of Hallopeau (ACH), a form of localized pustular psoriasis, remain poorly understood.^1^ Despite the availability of several treatment options, patients with ACH often have an insufficient response to therapy. No standardized guidelines, including disease severity score, have been developed for ACH management, and molecular profiling of ACH has yet to be established. Therefore, the development of more optimal and personalized therapeutic options with novel mechanisms of action is an urgent medical need. In this report, we evaluate the efficacy and molecular profile of anti–interleukin (IL)-23p19 (guselkumab [Tremfya, Janssen]) and anti–tumor necrosis factor-alfa (TNF-α) (certolizumab [Cimzia, UCB]) therapy for the treatment of severe ACH before and during pregnancy.

## Case report

**Fig 1:**
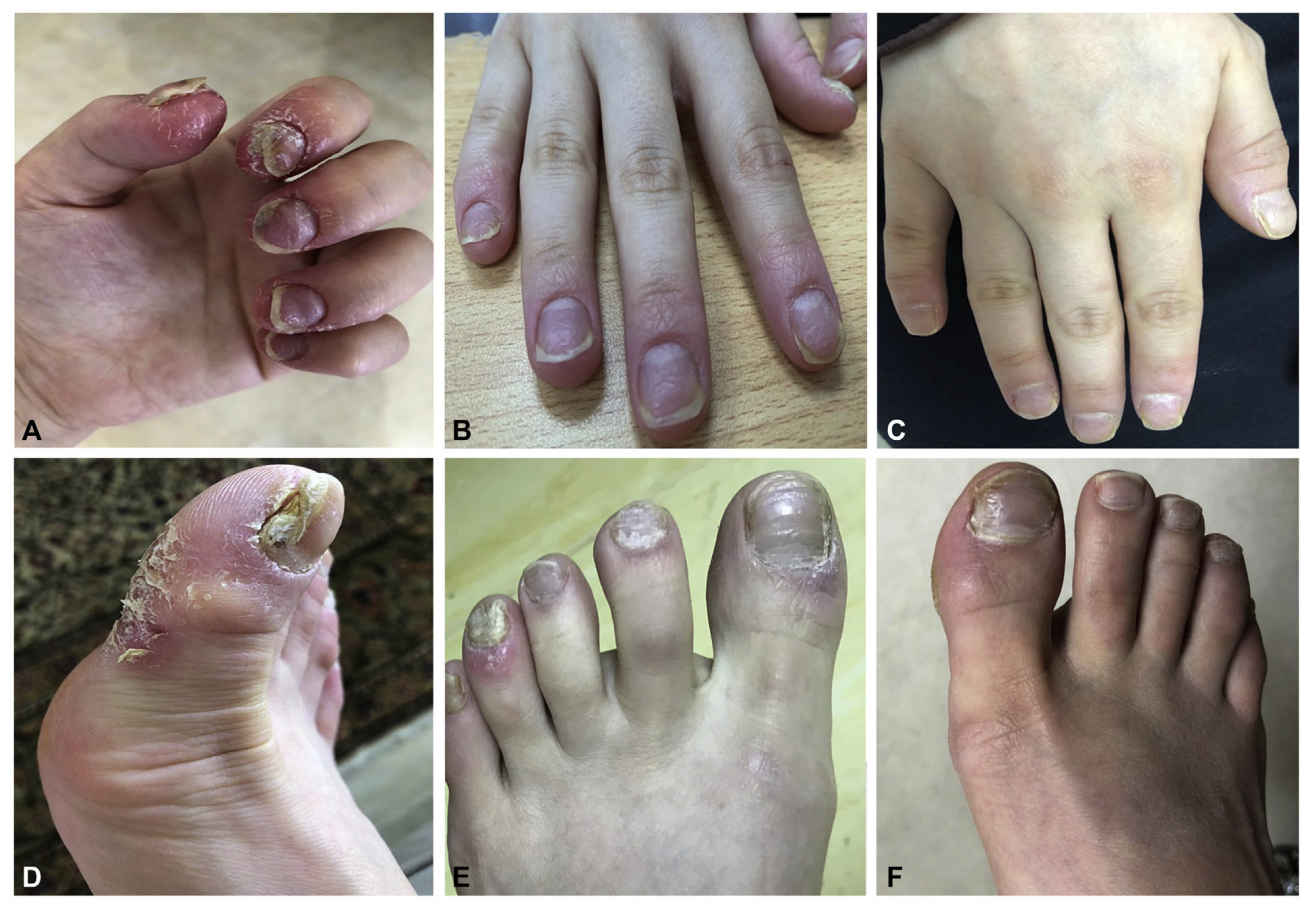
A 23-year-old woman with acrodermatitis continua of Hallopeau presented with marked erythema, edema, slight scaling, crusting, nail dystrophy, and severe pain involving all **(A)** fingernails and **(D)** toenails. **B** and **E**, Presentation after 12 weeks and **(C**, **F)** 36 weeks of certolizumab treatment.

A 23-year-old woman of the Arabian Peninsula origin presented with erythematous, edematous, and painful digits. The patient reported a pain score of 8 out of 10 in all 20 digits. Dermatologic examination revealed a severe erythemato-squamous, psoriasiform, pustular eruption surrounded by a hyperemic area affecting the terminal phalanges of all digits (Fig 1, *A* and *D*). The clinical findings supported the diagnosis of ACH. X-rays of the hands and feet did not reveal any bone or articular abnormalities. The results of gram stain and potassium hydroxide preparation were negative. Sanger sequencing did not reveal a mutation in ACH-associated *IL36RN* and *AP1S3* candidate genes.^2^, ^3^, ^4^, ^5^ All other routine laboratory findings were within normal ranges, and methotrexate therapy (25 mg weekly) was initiated. In a follow-up examination after 3 months, the patient showed only a minimal response to treatment with methotrexate. A class 4 topical steroid (clobetasol dipropionate) under occlusion and systemic prednisolone (1 mg/kg start, 0.5 mg/kg maintenance dose) were tried for 1 month without benefit. Considering recent publications promising success in the treatment of ACH with anti–IL-17 therapy, we initiated therapy with the IL-23 subunit α (p19 subunit) inhibitor guselkumab, which blocks both IL-23 and IL-17 release.^6^ Guselkumab was introduced according to the protocol for plaque psoriasis (100 mg subcutaneously). The patient had an excellent response (90% improvement) at week 12 (3 doses) (Fig 1, *B* and *E*). Guselkumab was continued with no adverse events, relapse, or need for additional treatment. During the 12th week of treatment, the patient reported being in the fourth week of pregnancy.

**Fig 2:**
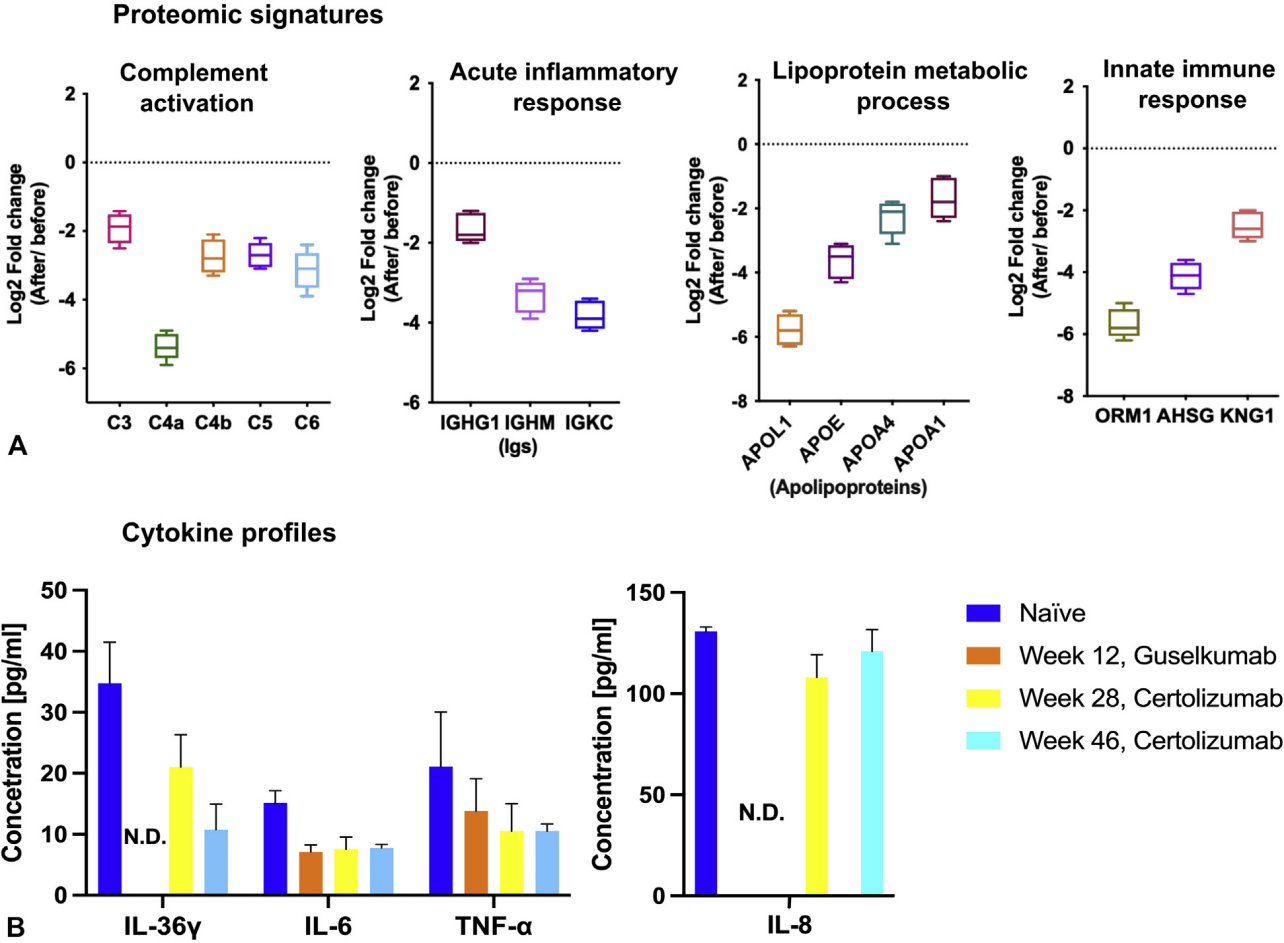
**A**, Proteomic signatures and **(B)** cytokine profiles of patient plasma during therapy. Box plots represent the fold change of differentially regulated proteins (naïve vs guselkumab) involved in different biologic processes. Proteins associated with active pathways in psoriasis were found to be significantly down-regulated (fold change range of −2.0 to −6.0). *AHSG*, Alpha-2-HS-glycoprotein (fetuin-A); *APO*, apolipoprotein; *IGHG1*, Ig gamma-1 chain C region; *IGHM*, Ig μ chain C region; *IGKC*, Ig κ chain C region; *IL*, interleukin; *KNG1*, kininogen-1; *N.D.*, not detected; *ORM1*, alpha-1-acid glycoprotein 1; *TNF*, tumor necrosis factor.

Therefore, guselkumab treatment was stopped, and because anti–TNF-α was the only Food and Drug Administration–approved biologic treatment of choice during pregnancy, certolizumab treatment was initiated. The patient continued to show remarkable improvement with respect to lesions and pain score (Fig 1, *C* and *F*) while receiving certolizumab (400 mg subcutaneously/every other week for 1 month, 400 mg subcutaneously/month maintenance). At 18-month follow-up, the patient had given birth without complications on the due date. During follow-up visits, she showed sustained improvement, no signs of active disease, and only discrete residual erythema. With the patient's agreement, it was decided to switch back to guselkumab, which produced an excellent response and reduced more effectively identified key disease-associated cytokines (Fig 2, *B*).

Cytokine and proteomics profiling of plasma samples confirmed the efficacy of the initiated antiinflammatory biologic therapy. Proteomics analysis identified therapy-dependent down-regulation of unique proteins belonging to inflammation-associated functional pathways, including complement activation, lipoprotein metabolism, and innate immune and acute inflammatory responses (Fig 2, *A*). Cytokine profiling showed a therapy-dependent decline in plasma levels of the immune response mediators IL-36-γ, IL-6, and TNF-α (Fig 2, *B*). Notably, IL-8 plasma levels were affected by guselkumab but not by certolizumab treatment (Fig 2, *B*).

## Discussion

ACH is a difficult-to-treat, poorly understood subtype of pustular psoriasis.^1^ Treatment of ACH patients with anti–TNF-α has had conflicting results. Adalimumab had a desirable effect in ACH.^7^ Although ustekinumab was reported to be effective in one study, the therapeutic dose had to be doubled, and complete control of ACH was achieved only after the addition of acitretin.^8^ Ustekinumab in combination with cyclosporine and prednisone did not result in clearance of a highly resistant form of ACH.^8^ Interestingly, retreatment with ustekinumab after 7 months of interruption resulted in a slower and poorer response than the initial regimen.^9^ It is important to note that assessment of therapeutic efficacy and comparison of different treatments is difficult due to the lack of a specific standardized severity score for ACH. However, a recent multicenter retrospective study found that TNF blockers and ustekinumab led to improvement in ACH and might demonstrate efficacy in a majority of cases.^10^ In contrast, certolizumab (anti-TNF) and guselkumab (anti-IL23p19) have been poorly studied for efficacy and safety in ACH. Here, we show that the TNF blocker certolizumab is an effective biologic agent for ACH during pregnancy. In addition, we show that guselkumab is a safe and effective biologic agent for ACH, targeting IL-23p19 without loss of efficacy and reducing important disease-associated cytokines (Fig 2, *B*). Our observations and molecular study add to the heterogeneity of the pathologic mechanisms of ACH and demonstrate the effects of guselkumab on molecular levels of inflammation in ACH, as well as demonstrating that proteomic profiling may be an important tool to stratify biologic therapy decisions.

## Conflicts of interest

Dr Steinhoff is a consultant for Pfizer, Janssen, Eli-Lilly, Novartis, UCB, Celgene, Galderma, Leo, Sanofi, Galderma, MenloTx. Sanofi, and Regeneron; has received grants from 10.13039/100004319Pfizer, 10.13039/100004336Novartis, Leo, and 10.13039/501100009754Galderma; and has been a speaker for Pfizer, Janssen, Eli-Lilly, Novartis, UCB, Celgene, Galderma, MenloTx, Sanofi, and Regeneron. Drs Al-Khawaga, Krishnankutty, Sher, Hussain, and Buddenkotte have no conflicts of interest to declare.
